# Antibiotics susceptibility pattern of Streptococcus pneumoniae isolated from sputum cultures of human immunodeficiency virus infected patients in Yaoundé, Cameroon

**DOI:** 10.11604/pamj.2018.31.16.11195

**Published:** 2018-10-05

**Authors:** Michel Kengne, Marlise Beatrice Bidzogo Lebogo, Julius Mbekem Nwobegahay, Bienvenue Etogo Ondigui

**Affiliations:** 1 School of Health Sciences, Catholic University of Central Africa, Yaoundé, Cameroon; 2 Yaoundé Military Hospital, Cameroon; 3 Yaoundé Central Hospital, Cameroon

**Keywords:** Antibiotic susceptibility, Streptococcus pneumoniae, lower respiratory tract infections, HIV patients

## Abstract

**Introduction:**

The susceptibility of Streptococcus pneumoniae to commonly used antibiotics is threatened by the emergence of resistance of S. pneumonia strains. So, to improve the management of lower respiratory tract infections (LRTIs) in human immunodeficiency virus infected patients, we assessed the antibiotic susceptibility of Streptococcus pneumoniae which is the most common bacterial cause of LRTIs in patients.

**Methods:**

A cross sectional study was carried out from May to October 2014. HIV infected patients suspected of LRTIs attending the Center Medical laboratory and those followed up at the authorized treatment center of Yaounde Military Hospital in Cameroon were enrolled. Sputum was collected from each patient and cultured; identification of microorganisms was performed following standard methods. The disk diffusion method was used for antibacterial susceptibility testing according to the Antibiogram Committee of French Society for Microbiology guidelines.

**Results:**

A total of 51 (25.5%) isolates of *S. pneumoniae* were recovered from sputum samples obtained from 200 HIV infected patients aged 19-66 years old (mean age: 36±10.087 years old); 144 (72%) of them were female (sex ratio M/F: 1/3). *S. pneumoniae* carriage was not age dependent (P = 0.384) and was significantly higher in male compared to female (P = 0.008). *S. pneumoniae* isolates were susceptible to amoxicillin-clavulinic acid (100%), pristinamycin (100%), erythromycin (100%) and cefixime (98.04 %). Highest resistance rates were recorded with fusidic acid (100%), fosfomycin (100%) and tetracyclin (100%).

**Conclusion:**

*S. pneumoniae* is still susceptible to some agents in our study area however; ongoing surveillance for antimicrobial susceptibility remains essential to identify emerging resistance and attempt to limit its spread.

## Introduction

Studies have shown that bacterial infections represent an important cause of morbidity and mortality in HIV infected patients [1-3]. The most causative organisms are Streptococcus pneumoniae and Haemophilus influenza [4, 5]. *S. pneumoniae* has been reported to be the most common bacterial cause of lower respiratory tract infections [6]. In countries where HIV epidemic remains entrenched, the dominance of LRTIs as the most common cause of hospitalization and mortality in HIV infected patients has been described [6]. These high-risk patients have reduced ability to eliminate microbial pathogens, and so outgrowth of resistant mutants during antibiotic treatment is more likely to occur [7-9]. Moreover it has been described elsewhere that widespread antibiotic resistance is common and seems higher in immune-compromised subjects than in immune-competent individuals [10]. The steady increase of bacteria resistance to antibiotics is a cause of global concern. Infections caused by resistant microorganisms often fail to respond to empiric treatment resulting in prolonged illness and greater risk of death [11]. Available studies from sub-Saharan countries in Africa have highlighted unexpected high rates of resistance of bacteria to common antibiotics [12-18]. In Cameroon, health institutions have little to none antibiotic policy. In addition, published data on LRTIs pathogens and their antibiotic resistance profiles in patients infected with HIV are lacking.

## Methods

**Patients:** A cross sectional study was carried out from May to October 2014. A total of 200 HIV infected patients with suspected LRTIs attending the Center Medical laboratory and those followed up at the authorized treatment center of Yaounde Military Hospital in Cameroon were eligible for inclusion. Patients who had received antibiotics within the previous fifteen days were excluded.

**Bacterial isolation, identification procedures and antimicrobial susceptibility testing:** Patients were instructed to collect deep coughed sputum into a sterile wide mouth container with a screw cap after rinsing the mouth twice with plain water. The samples were brought immediately to the laboratory and processed within 30 minutes of collection. Sputum samples were inoculated onto blood agar plate and incubated in a candle jar for 24h. *S. pneumoniae* was identified using gram stain, catalase and optochin tests that were performed in parallel. The antimicrobial susceptibility of the isolated bacterium was determined using the disc diffusion method as described by the Antibiogram Committee of French Society for Microbiology [19]. The following antibiotics purchased from Bio-Rad (Belgium) were used: amoxicillin/ clavulanic acid (20/10 μg), imipenem (10μg), oxacillin (5μg), cefixime (10 μg), pristinamycin (15μg), tetracyclin (30UI), ofloxacin (5 μg), erythromycin (15UI), fusidic acid (10 μg), fosfomycin (50μg) and rifampicin (30 μg). The reference strain used for quality control was *S. pneumoniae* CIP 104485.

**Data analysis:** Data were entered and analyzed using SPSS version 12.0 for windows (SPSS, Inc., Chicago, IL). Discrete variables were expressed as percentages and proportions, and then compared using the Chi-square test. Statistical significance difference was considered at value of p<0.05.

**Ethics:** Authorization to conduct this study was obtained from the Cameroon national ethical review committee. Informed consent was obtained from all participants ≥ 18 years; while assent consent was obtained from participant teenagers via a proxy.

## Results

From May to October 2014, a total of 200 sputum samples obtained from 200 HIV infected patients were processed. The mean age at presentation was 36±10.087 years (range 19-66 years). There were 144 (72%) females and 56 (28%) males. The male to female ratio was approximately 1:3. *S. pneumoniae* was found in 51 (25.5%) of the cases. *S. pneumoniae* carriage was not age dependent (P = 0.384) and was significantly higher in male compared to female (P = 0.008) as shown in Table 1. Amoxicillin/clavulanate (100%), erythromycin (100%), pristinamycin (100%) and cefixime (98.0 %) displayed satisfactory activity against the isolates. 100% of the isolates were resistant to fusidic acid, fosfomycin and tetracyclin as shown in Table 2.

**Table 1:** Distribution of *S. pneumoniae* isolates based on patient age group and sex

Antibiotics		*S. pneumonia* isolates (No= 51)
Betalactamines	Amoxicillin/clavulanate	100
	oxacillin	78.4
	Cefixime	98.0
Fluoroquinolones	ofloxacin	88.2
Tetracyclines	tetracyclin	0
Macrolides	Erythromycin	100
Streptogramines	pristinamycin	100
Others	Fusidic acid	0
	Fosfomycin	0
	Rifampicin	35.3

**Table 2:** Percentage of susceptibility of *S. pneumoniae* isolates to antibiotics tested

	No of total cases	No of total positive cases	Percentage	Statistical significance
**Age group of patients**				
15-29	64	09	14.06	
30-44	94	31	32.97	χ^2^=22.28 p= 0.384
45-59	32	07	21.80	
60-74	10	04	40	
Total	200	51	25.5	
**Sex of patients**				
Male	56	17	30.35	χ^2^=18.95 p= 0.008
Female	144	34	23.61	
Total	200	51	25.5	

## Discussion

Since local susceptibility patterns are essential for antimicrobial prescribing, this work aimed at determining the antibiotics susceptibility patterns of *S. pneumoniae* isolated from sputum cultures of human immunodeficiency virus infected patients in Yaoundé-Cameroon. In the current study, the rate of carriage for *S. pneumoniae* was 51/200 (25.5%). This result is different from that of other studies. Pemba et *al*. [20] obtained a prevalence of 8.8% (n=856) among HIV-infected mineworkers in South Africa whereas in the same country, Crewe-Brown et *al*. [21] recorded a prevalence of 15.5% (n=457) from patients with and without human immunodeficiency virus infection. This finding may indicate the advanced impairment of the immune system defense mechanism of the study population which confer them a reduced ability to eliminate microbial pathogens. However, CD4+ T-cell counts and viral load measurements of the patients were not examined. The present investigation found that male to female ratio for *S. pneumoniae* carriage was approximately 1.3:1. This result is in line with other published data where a male to female ratio of ~1.5-2:1 was seen in most studies of pneumococcal disease. The predominance of male for *S. pneumoniae* infection may be due to underlying conditions such as smoking and alcoholism which are more common among males [22]. The resistance of *S. pneumoniae* to β-lactam agents and other antimicrobial agents is increasing in many parts of the world [23, 24].

According to Friedland and McCracken [25], all β-lactam antibiotics act by interaction with penicillin binding proteins, and changes in these proteins result in decreased susceptibility to all antibiotics of this type. Our study showed that nearly all *S. pneumoniae* isolates were susceptible (> 90%) to β-lactamine agents. The high susceptibility of the isolates may be due to the appropriate use of this agent in treating *S. pneumoniae*-related diseases. The same conclusion may be drawn for macrolide agent (erythromycin) since all the isolates were susceptible (100%). A worldwide increase of the outbreak of macrolide resistance in *S. pneumoniae* among clinical isolates has been reported by Najafi Mosley et *al*. [26]. 88.2% of the isolates were susceptible to ofloxacin. This observation is in line with the results published by karlowsky et al. [27]. In the opposite, it has been reported that pneumococci have borderline susceptibility at the recommended dosages to the earlier generation of fluoroquinolones such as ofloxacin and therefore are not recommended for the treatment of pneumococcal infections [25, 28]. The increasing prevalence of pneumococci with reduced susceptibility to fluoroquinolones has been assigned to the selected pressure of resistant mutants due to the increased use of fluoroquinolones [29]. In the present study, all the isolates were resistant to fusidic acid, fosfomycin and tetracycline, probably as a result of the exposure of the study population to other antibiotics. According to previous studies [20, 30], many HIV-positive patients are given primary cotrimoxazole (trimethoprim/sulphamethoxazole) prophylaxis and exposure to this antibiotic might increase the risk of antibiotic resistance in a variety of bacterial pathogens that may infect this high-risk population.

## Conclusion

Amoxicillin/clavulanate, erythromycin, pristinamycin and cefixime are still active antibacterial agents and therefore the drugs of choice in treating *S. pneumoniae*-related diseases in our setting. More studies like this are required at regular interval, to formulate an antibiotic policy which helps in preventing mortality and morbidity due to LRTIs caused by this bacterium in immune-compromised individuals.

### What is known about this topic

- *S. pneumoniae* is the most important cause of morbidity and mortality in HIV infected patients;
- Antibiotics resistance is common in immune-compromised subjects;
- Infections caused by resistant microorganisms often fail to respond to empiric treatment.

### What this study adds

- Knowledge of S. pneumonia carriage among HIV+ patients in Cameroon;
- Information about the antibiotics to be used in treating *S. pneumoniae* infection among HIV+ patients in the study area.

## Competing interests

The authors declare no competing interests.
